# Oral dysbacteriosis in type 2 diabetes and its role in the progression to cardiovascular disease

**DOI:** 10.4314/ahs.v17i4.16

**Published:** 2017-12

**Authors:** Ziad Nabee, Rajesh Jeewon, Prity Pugo-Gunsam

**Affiliations:** 1 Department of Biosciences, Faculty of Science, University of Mauritius, Reduit, Mauritius; 2 Department of Health Sciences, Faculty of Science, University of Mauritius, Reduit, Mauritius

**Keywords:** Oral ecology, saliva, bacteria, dental caries, diabetes

## Abstract

**Background:**

Salivary changes and proliferation of specific bacterial communities are known to result in oral disease which may adversely impact on systemic conditions like diabetes and cardiovascular diseases.

**Objectives:**

This study reports on the changes in oral ecology of healthy and diseased adults and the possible role in disease causation.

**Methods:**

The study comprised 150 participants divided into control (healthy), diabetic and cardiac groups. After dental examination for (Decayed Missing Filled Teeth (DMFT) and Oral Rating Index (ORI), stimulated saliva was sampled to determine flow rate and buffering capacity. Salivary microbial load of *Streptococcus mutans* and *Lactobacilli* were subsequently quantified.

**Results:**

DMFT, ORI, buffering capacity and flow rate were inferior for both diabetic and cardiac patients, who had higher bacterial counts (p<0.05). Long standing diabetics harboured a higher load of *Streptococcus mutans*. The microbial load of *Streptococcus mutans* in cardiac patients was double that of diabetics.

**Conclusion:**

Disruption in the salivary environment and changes in microbial ecology with increased load of cariogenic bacteria were found in diabetic and cardiac patients. This study brings forward new evidence of a markedly higher load of *Streptococcus mutans* in cardiac patients which may underlie the progression of diabetes to cardiovascular disease in this population.

## Introduction

Oral health is dependent on the maintenance of stable microbial communities and oral disease occurs when pathogenic species outgrow the normal flora^1^. In the oral cavity, pathogenic bacteria are often associated with two major diseases; dental caries and periodontal disease^2^. Saliva, which constantly bathes the oral cavity reflects an imbalance in microbial communities under these disease conditions^3^. In dental caries, there is a shift towards community dominance by acidogenic and acid-tolerating species such as *Streptococci mutans* and *Lactobacilli*^4^. Among the 200 bacterial species isolated from dental plaque the two most pathogenic for dental caries are: *Streptococcus mutans*^5^ and oral *Lactobacillus*^6^. These bacteria are documented to produce organic acids in the oral cavity and are able to maintain its metabolism in strong acid medium, rendering them cariogenic^7^. In the mouth, salivary imbalances in flow rates and buffering capacity have been shown to impact on the development of dental caries^8^,^9^. The dynamics involved in dental caries progression, compounded by resulting inflammation produced by these cariogenic bacteria often lead to periodontal disease in subjects afflicted with dental caries.

Oral bacteria have long been hypothesised to participate in formation and aggravation of chronic non-communicable diseases (CNCDs) such as diabetes mellitus and cardiovascular diseases amongst others^2^. In Mauritius, these two CNCDs are the principal underlying causes of mortality, accounting for 1,545 (17.3%) and 2,277 (25.4%) deaths respectively^10^. A review of the literature indicates that few studies have reported on cariogenic bacterial load among diabetics and none in subjects with cardiovascular disease. Studies conducted in diabetic children has until now shown a lack of correlation between salivary levels of *Streptococcus mutans, Lactobacilli* and dental caries^11^. Data concerning oral health in Mauritius is very scant. Public health statistical figures with respect to oral health are recent and depict a gradual soar in the number of tooth fillings and restorations. From 2006 to 2011, dental attendance at public health services has increased by 10%^10^.

Despite the fact that *Streptococcus mutans* and *Lactobacilli* have both been associated with the presence and progression of dental caries, the relation between these pathogens and oral health especially in diseased Mauritian subjects is unclear. In 2011, the first local study investigating the oral microbial population in diseased Mauritian adults confirmed the implication of *Lactobacilli* and *Streptococcal* species^12^. This present study takes this a step further by quantifying the degree of the ecologic changes in the oral cavity of diseased subjects while identifying other possible factors such as salivary properties and oral hygiene, which may be implicated. The main objectives were to investigate differences in biochemical characteristics and cariogenic microbial load of saliva between diabetics and cardiac subjects. Moreover, the extent to which these parameters impacted on oral and general health conditions were also determined.

## Material and methods

### Subjects and study design

The subjects were 150 Mauritian adults aged 25 to 65 years. The sample included equal numbers of healthy controls (n=50), diabetics (n=50) and diabetics with cardiovascular disease (n=50). Smokers, subjects wearing dentures, individuals who were undergoing antibiotic or other anti-microbial therapy within 3 months prior to the examination and those suffering from any other ailment besides diabetes and cardiovascular disease were excluded from the study. Non-insulin dependent diabetics were chosen under this study. All participants received verbal and written information about the study, and signed consent forms prior to participation. Patients were assured of confidentiality and informed of their right to refuse to participate or withdraw from the study. The study protocol was approved by the Ethics Committees of the Ministry of Health and Quality of Life, Mauritius and the University of Mauritius Ethics Committee. All experimental procedures were in accordance with the standards of both committees and the Helsinki Declaration of 1975, as revised in 1983. A short questionnaire was used to elicit information from participants. The number of years of disease affliction was recorded followed by questions on oral hygiene practices. These questions had set answers and included the number of times participants brushed their teeth daily, the time taken to brush their teeth, whether any other dental hygiene methods were used, and also whether patients attended dental checkups regularly which allowed subjects to be categorised as having (i) poor oral hygiene (ii) Average oral hygiene and (iii) good oral hygiene^13^.

### Oral health examination

Standardised oral health examination was conducted for each participant by qualified dental practitioners after teeth were air-dried, under artificial light and with the aid of a dental mirror and explorer^14^. Cotton rolls were used to control salivary flow. Oral health status was assessed by measuring the Decayed, Missing, and Filled teeth (DMFT) which is well established as a leading measure of dental caries experience in epidemiology^15^. In addition to the DMFT index which quantifies oral health in terms of dental caries, periodontal condition was assessed by the Oral Rating Index (ORI) which varies from 2 to -2. The ORI which presents the advantages of being a quick and reliable method for recording of periodontal status, informs on gingival condition, calculus and plaque accumulation^16^.

### Salivary sampling and salivary flow rate measurement

After oral examination, stimulated saliva samples were collected. Subjects were given thorough instructions beforehand regarding the procedures pertaining to saliva collection. They were also told not to eat or drink at least 2 hours before sampling. After swallowing pre-existing saliva, subjects were given 1 gram of an inert elastic and instructed to masticate at a constant rate of about 50 to 70 strokes per minute^17^. After each 30 seconds, participants spitted out into a graduated salivette. Salivary collection lasted for 5 minutes and the salivary flow rate was defined as the volume of saliva secreted per minute. By means of a sterile pipette, 1 ml of collected saliva was transferred to sterile tubes containing 4 ml of peptone water. The samples were immediately transported in an ice box to the research facility and processed accordingly.

### Buffering capacity

Salivary pH was determined immediately after collection of the unstimulated saliva samples in order to avoid any time-related pH changes or loss of carbon monoxide. The buffering capacity of saliva was assayed by measuring 0.5 ml of saliva, which was added to 1.5 ml of 5 mmol/L HPLC grade hydrochloric acid (Fisher Scientific, Cheshire, UK). The mixture was vigorously shaken and centrifuged at 2500rpm for 1 minute^18^. It was allowed to stand for an additional 10 minutes before the final pH was measured (Hannah Instruments, Rhode, US).

### Bacteriological profiling

The saliva/peptone sample was vortexed using a cyclomixer (Stuart Scientific, UK). A 1µL loop was used to inoculate the 1:5 diluted sample on Mitis Salivarius Bacitracin (MSB) Agar and Rogosa SL (RSL) Agar (Oxoid, Hampshire, UK). The MSB and RSL were both incubated anaerobically at 37°C for 48 hours and 96 hours respectively^19^. Following incubation, counts were made of colonies exhibiting morphological characteristics of *Streptococcus mutans* and *Lactobacilli*. Bacterial colonies were deduced on colony appearance, gram staining, and biochemical testing as well as carbohydrate fermentation potential followed by bacterial counts. Further confirmatory testing for *Streptococcus mutans* was performed by colony sub-cultures on blood agar to assess haemolytic potential. Colony counts were expressed as colony forming units per milliliter (CFU/mL) of saliva. Semi-quantitation of the number of colonies was done by correcting the actual colony count for the dilutions made upon sampling^20^.

## Statistical analyses

SPSS, version 16.0 (SPSS Inc., Chicago, IL, USA) was used to conduct the statistical analyses. An alpha level of 0.05 was used. One-way analysis of variance was used to determine any significant difference among the three groups for oral hygiene practice scores, DMFT scores, salivary parameters and microbial load. Post hoc analysis was carried out to examine the significance of relationships between and within groups. Pearson's correlation analysis was used to study the relationship between salivary parameters and DMFT. Multiple regression analysis allowed assessing multifactor impact on oral health while controlling for oral hygiene of the subjects.

## Results

### Oral Hygiene Practice Score, DMFT and ORI

No significant difference was seen in Oral Hygiene Practice between the diabetic, cardiac and control groups (p>0.05). Approximately two thirds of the subjects observed average oral hygiene practices and one third observed poor oral hygiene practices independent of the three groups. One way ANOVA showed that Decayed, Missing, and Filled teeth (DMFT) scores were significantly different for the three groups (F=35.295, p<0.05). The control population (healthy) showed a mean of 7.6±3.81 as compared to 11.6±3.49 for diabetics and more than a double increase (16.1±6.23) for cardiac subjects (Table 1). DMFT scores were significantly different between the three groups (p<0.05).

**Table 1:** Mean DMFT scores of subjects

Subjects	Decayed Missing Filled Teeth (DMFT) Score	Statistical values
Healthy	7.6 ± 3.81	P<0.05
Diabetic	11.6 ± 3.49	
Cardiac	16.1 ± 6.23	

**Figure 1:**
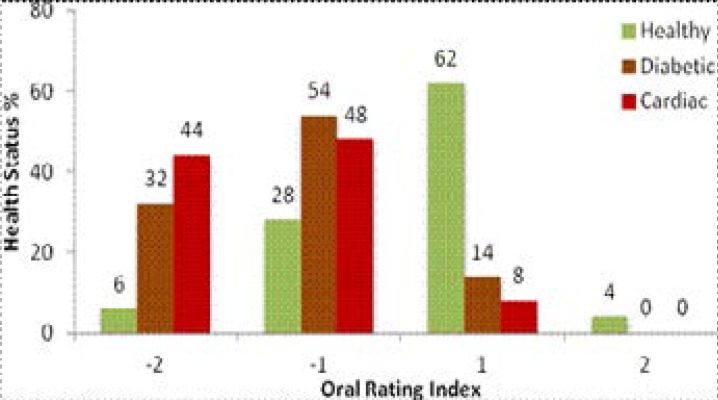
Percentage subjects based on Oral Rating Index (ORI) scores

Similar observations were recorded for the Oral Rating Index. Oral examination showed that the ORI score dropped from 2 to -2 from healthy to diabetic subjects and further to the cardiac participants (p<0.05). Approximately one-third of them had positive scores, while the rest observed negative scoring with the majority of subjects falling in the (−1) category. In the cardiac group, 48% of subjects had an ORI score of −1 and 44% of the same group were positive for periodontitis compounded by very poor oral hygiene. Overall, 32% of diabetics suffered from periodontal disease (Figure 1).

### Salivary flow rate, pH, buffering capacity and DMFT

The control group had significantly different flow rate (1.9±0.19 mL/min) from the diabetic and cardiac groups which were 1.7±0.26 mL/min and 1.7±0.22 mL/min respectively (Table 2). No significant difference was observed between the diabetic and cardiac groups (LSD, p>0.05). A significant association was further noted between flow rate and Decayed, Missing, and Filled teeth (DMFT) index (p<0.05).

**Table 2:** Mean Salivary Flow Rate (SFR) of subjects

Health Status	Salivary Flow Rate (SFR) mL/min	Statistical values
Healthy	1.9 ± 0.19	P<0.05
Diabetic	1.7 ± 0.26	
Cardiac	1.7 ± 0.22	

The control group had a mean pH of 7.41±0.39 compared to 6.21±0.53 for diabetics and 5.87±0.53 for cardiac subjects. The salivary buffering capacity followed a similar trend (Table 3). The mean pH and buffering capacity values were significantly different between groups as given by ANOVA and Post Hoc Analyses (p<0.05). Multiple regression revealed that salivary pH and buffering capacity together correlated strongly to the DMFT index (r2=0.48, p<0.05).

**Table 3:** Mean pH and buffering capacity of subjects

Health status	pH	Buffering capacity	Statistical values
Healthy	7.4 ± 0.39	5.4 ± 0.25	P<0.05
Diabetic	6.2 ± 0.53	4.4 ± 0.48	
Cardiac	5.9 ± 0.53	3.9 ± 0.45	

### Microbial load of streptococcus mutans and Lactobacilli, DMFT and ORI

**Figure 2:**
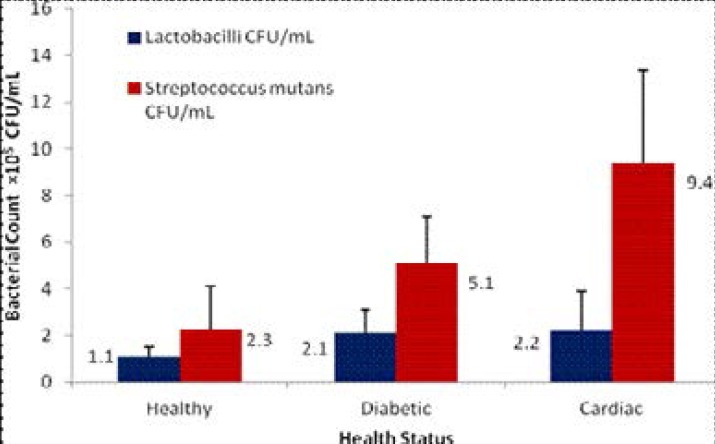
Mean Colony Counts in CFU/mL

All salivary samples cultured yielded microbial growth at different colony counts. 3.3% of the cultures yielded no growth of *Streptococcus mutans* while 8% of the samples yielded no growth of *Lactobacilli*. Correction with appropriate dilutions resulted in *Lactobacilli* counts of 1.1×10^5^ (SD±0.42) CFU/mL for the control group, 2.1×10^5^ (SD±1.01) CFU/mL for diabetics and 2.2×10^5^ (SD±1.70) CFU/mL for the cardiac group (Figure 2). However, in respect of *Lactobacilli* counts, there was statistically no significant difference between the diabetic and cardiac groups (p>0.05). In respect of *Streptococcus mutans* counts, microbial load recorded for the control group was 2.3×10^5^ (SD±1.79) CFU/mL. The count in diabetics was 5.1×10^5^ (SD±1.99) CFU/mL, double that of the controls. The cardiac group had a load of 9.4×10^5^(SD±3.98) CFU/mL, showing approximately 400% increase as compared to the controls and a count double that of diabetics.

Differences between groups were significant for bothcariogenic bacterial counts (p<0.05). Multiple regression analysis showed that the count of *Streptococcus mutans* was significantly associated with oral health despite controlling for oral hygiene (p<0.05). The microbial load of *Streptococcus mutans* significantly correlated with Decayed, Missing, and Filled teeth (DMFT) scores (r2=0.54, p<0.05) and the Oral Rating Index (r2=0.49, p<0.05). No association was found between *Lactobacilli* count and oral health status. The microbial load of *Streptococcus mutans* was further correlated to the number of years subjects were afflicted with the diabetic condition. Statistically significant associations were found as the microbial load increased with long standing diabetes (p<0.05).

## Discussion

### Oral hygiene practice score and oral health

It is well recognised that good oral hygiene impacts significantly on oral health status by translating into lower levels of dental caries^21^,^22^. However, data is scarce for those suffering specifically from diabetes and cardiovascular disease as compared to healthy controls. In this study, evaluation of oral hygiene practices revealed that good oral hygiene was not practised among any of the three study groups (control, diabetic and cardiac). No significant difference was observed between oral hygiene practices among diabetic and cardiac subjects when compared to the control group (healthy). Similar behaviours have previously been noted in diabetics where suboptimal oral hygiene practices were observed^23^. Despite the fact that diabetic patients require more intensive oral hygiene practice, such trend is not observed in this study. The diabetic condition compounded by suboptimal oral hygiene behaviours ultimately leads to increased dental caries. As expected, Decayed, Missing, and Filled teeth (DMFT) scores for the control group were significantly lower than diabetics and cardiac patients (Table 1) indicating that poor oral health was more prevalent for diabetics and further for diabetics with cardiovascular disease.

Our results corroborate with the findings of a comparable study in Malaysia where non-insulin dependent diabetics had significantly higher mean DMFT values (13.52 ± 3.694) as compared to healthy controls (9.73 ± 2.496)^24^. Our results are also is agreement with earlier reports where the mean DMFT scores of diabetics were observed to be higher than case matched healthy controls^11^,^25^,^26^. Other studies have also found that patients with diabetes are more susceptible to oral sensory, periodontal, and salivary disorders, which could increase their risk for developing new and recurrent dental caries^27^. Contrary to our findings, several studies have shown that the DMFT scores of diabetics are either similar to or lesser than that of non-diabetics^28^–^32^. Their findings have been attributed to the fact that caries is more strongly related to diet and also because of similar salivary flow rates documented in diabetics and non-diabetics. Nonetheless, in this study, the high DMFT scores observed for diabetics may also be due to salivary hyperglycaemia which is documented to manifest in the diabetic condition, directly increasing risk of dental caries^33^. The fact that most of the cardiac patients in Mauritius are initially diabetic may explain the higher DMFT scores recorded in this respect. However, to date, no comparative data is available as far as cardiac patients are concerned.

The Oral Rating Index followed similar trends as the DMFT index. Gingivitis, poor oral hygiene and periodontal disease which were significantly higher in diabetics and cardiac were also observed by a previous study in diabetic subjects^34^. Our results also corroborate with high presence of gingivitis and periodontal disease observed in similar studies^35^,^36^,^37^. In addition, a case control study in Indian adults showed that besides high DMFT scores, patients with coronary heart disease had an increase in mild periodontal disease as compared to healthy controls. The similarity of results observed in Indian countries with our findings may be related to the fact that Mauritians are mostly of Indian descent. This alludes to culture, which may be the driving factor, specifically via high carbohydrate diets, which is common for both countries and which provides with fastidious sources of nutrition for the proliferation of oral cariogenic bacterial species. The higher occurrence of gingivitis and high level of dental caries have also previously been observed in cardiac patients from India^38^.

### Salivary flow rate, buffering capacity and oral health

As documented in previous reports^39^,^40^, saliva plays a key role in maintaining oral health and serves as an aid to oral diagnosis via flow rate and composition which are recognized as important markers against dental caries^41^. In this study, the salivary flow rate decreased significantly from healthy controls to the diseased groups. This is in accordance to findings published in research studies involving type 1 and type 2 diabetics^42^,^43^. The lower salivary flow rates can be attributed to the use of prescription medications in patients as all diabetics patients under study were non-insulin dependent^42^. However, systemic conditions (e.g diabetes) may also directly affect the functional capacity of salivary glands resulting in decreased flow rate^27^. Consequently, the decreased salivary flow in diabetics heavily impacts on oral health^44^. Another major finding was that the stimulated salivary flow rate was significantly associated with the Decayed, Missing, and Filled teeth (DMFT) scores recorded. A decrease in salivary flow rate translated into an increase in DMFT scores, documented in literature to alter saliva composition, ultimately increasing risk of dental caries^45^. It has also been reported that the decreased salivary flow rates in patients with cardiovascular disease may be attributed to low-dose aspirins and statins often used during treatment of these patients^46^. However, our study shows that there is no significant difference between salivary flow rates of diabetic and cardiac subjects.

Biochemical markers such as salivary pH and buffering capacity were significantly different between groups decreasing from healthy to diabetic and further to cardiac subjects (Table 3). These findings are in agreement with a previous study which reported lower pH of whole saliva in respect of diabetic subjects which subsequently translated into reduced buffering capacity^47^. Our results reflect similar findings as in other previous studies evaluating the protective role of salivary factors like pH, salivary flow rate and buffering capacity on oral health^48^–^50^. Since the salivary pH is directly related to the flow rate, reduction in the salivary flow rate directly leads to changes in pH^46^. The salivary pH and buffering capacity also significantly correlated with DMFT scores (r2=0.48). Our results also confirm the findings of previous studies^3^,^51^,^52^, which showed that reduced level of salivary pH in subjects correlated with higher levels of dental caries.

### Microbial profiling and oral health

Of the cariogenic oral bacteria, *Lactobacilli* and *Streptococcus mutans* have the most prominence as far as dental caries are concerned^53^,^54^. The high bacterial loads observed in both diabetic and cardiac subjects corroborate with a recent study wherein elevated numbers of culturable *streptococci* and *lactobacilli* were obtained in similar patients^55^,^56^. To date, few studies in literature have researched and compared the bacterial profiles of saliva in diabetic patients and cardiac patients. In this study, the *Streptococcus mutans* count was significantly associated with the oral health of the participants (p<0.05) and strongly correlated with the Decayed, Missing, and Filled teeth (DMFT) Scores (r2=0.54) as well as the Oral Rating Index (r2=0.49). This concurs with a similar report which found a positive association between the number of carious teeth with higher levels of *Streptococcus mutans* and *Lactobacilli* among diabetic subjects^57^. In addition, a significant association was observed between number of years of affliction and a superior load of *Streptococcus mutans* which supports the increased inflammation documented to be present in the oral cavity of diabetics^58^.

Despite the fact that *lactobacilli* species were found to be present in similar loads in both diabetic and cardiac patients, the bacterial load of *Streptococcus mutans* provided positive results which showed that its abundance in cardiac patients was almost double that in diabetic patients. It is thus possible that the high proliferation of *Streptococcus mutans* noted in cardiac patients put them at more risk of developing oral bacterial infections and ensuing systemic diseases^2^. However, it is important to point out that systemic conditions also weaken and alter physiological balance leading to oral dysbacteriosis and disease^59^. This is reflected in diabetic women whereby conditions of vaginal candidiasis worsen with hyperglycemia^60^. As well, this study shows that the non observance of recommended oral hygiene practices compounded by the omnipresent salivary hyperglycaemia in diabetic patients also provides with a suitable environment for the proliferation of specific oral bacteria. A key limitation to this study is the sampling size, which reflects the tedious microbiological work involved in this research. Moreover, the impact of dietary factors on microbial proliferation was not addressed in this research work.

## Conclusion

The main implication of the present study is that diabetic and cardiac subjects suffered from poor oral health with drastic changes in the oral environment as far as salivary properties and cariogenic bacterial load are concerned. The altered salivary composition presents as an important factor that serves to modify the proportions of specific oral bacterial species, ultimately predisposing to cariostatic lesions. In addition, our study brings forward new evidence that long standing diabetics harbour increased bacterial load of *Streptococcus mutans*. This indicates that the common progression of diabetics to cardiovascular disease in Mauritius may well be mediated by specific inflammatory mechanisms modulated by specific pathogenic oral bacterial species. This is further supported by the markedly higher microbial load of *Streptococcus mutans* in cardiac patients. The drastic alteration in pathogenic oral bacterial populations therefore may directly constitute a chronic source of systemic challenge. However, a causal relationship will only be confirmed by studying the behaviours of live oral pathogenic bacteria in cardiac tissues which requires invasive, costly and life threatening investigations. Likewise, it would also be important to consider the bidirectional relationship between bacterial infections and chronic systemic conditions. This study warrants the need for in-depth research using larger sample populations.
